# Supplementary material for: Vitamin D Metabolite Ratio in Pregnant Women with Low Blood Vitamin D Concentrations Is Associated with Neonatal Anthropometric Data
Source: Nutrients. 2022 May 25;14(11):2201. doi: 10.3390/nu14112201 (PMC9182679; doi:10.3390/nu14112201)
Supplement: Supplementary file 1 [file nutrients-14-02201-s001.zip › nutrients-1688823-supplementary.pdf]

Supplementary TableS1. Quality Control (QC) sample measurement values**.**

Compound	QC (SRM972a)	Expected conc. (ng/mL)	Average (ng/mL)	SD	CV (%)	Minimum (ng/mL)	Maximum (ng/mL)
$25(OH)D_3$	Level 1	28.8 ± 1.1	29.7	1.01	3.41	27.8	31.6
	Level 2	18.1 ± 0.4	18.5	0.68	3.66	17.9	20.1
	Level 3	19.8 ± 0.5	19.9	0.84	4.24	18.8	22.0
	Level 4	29.4 ± 0.9	29.5	1.27	4.30	27.7	32.1
3-*epi*-$25(OH)D_3$	Level 1	1.84 ± 0.08	1.80	0.07	3.92	1.70	1.89
	Level 2	1.29 ± 0.06	1.24	0.06	5.06	1.12	1.37
	Level 3	1.18 ± 0.13	1.07	0.07	6.41	0.95	1.21
	Level 4	26.4 ± 2.10	25.9	1.12	4.32	23.6	28.3
$25(OH)D_2$	Level 1	0.54 ± 0.06	0.51	0.03	6.52	0.46	0.59
	Level 2	0.81 ± 0.06	0.88	0.04	4.25	0.83	0.94
	Level 3	13.3 ± 0.30	14.1	0.65	4.61	13.2	15.3
	Level 4	0.55 ± 0.10	0.51	0.05	10.3	0.45	0.59
$24,25(OH)_2D_3$	Level 1	2.66 ± 0.10	2.57	0.11	4.47	2.43	2.80
	Level 2	1.41 ± 0.05	1.37	0.08	5.85	1.27	1.52
	Level 3	1.62 ± 0.06	1.57	0.06	4.09	1.49	1.67
	Level 4	2.64 ± 0.09	2.16	0.08	3.60	2.49	2.74

SD, standard deviation; CV, coefficient of variation
